# Effect of Industrial Processing on the Volatile Organic Compound Fingerprint of Dry-Cured Tuna

**DOI:** 10.3390/foods14040592

**Published:** 2025-02-11

**Authors:** Mónica Sánchez-Parra, Annalaura Lopez, Vittorio Maria Moretti, José Luis Ordóñez-Díaz, José Manuel Moreno-Rojas

**Affiliations:** 1 Department of Agroindustry and Food Quality, Andalusian Institute of Agricultural and Fisheries Research and Training (IFAPA) Alameda del Obispo, Avda. Menéndez–Pidal s/n., 14004 Córdoba, Spain; 2 PhD Program Ingeniería Agraria, Alimentaria, Forestal y del Desarrollo Rural Sostenible, Universidad de Córdoba, 14004 Córdoba, Spain; 3 Department of Veterinary Medicine and Animal Science (DIVAS), Università degli Studi di Milano, Via dell’Università 6, 26900 Lodi, LO, Italyvittorio.moretti@unimi.it (V.M.M.)

**Keywords:** volatile organic compounds (VOCs), yellowfin tuna, dry-cured tuna, fish flavour, SPME, ML-sPLS-DA

## Abstract

Dry-cured tuna products exhibit unique aroma characteristics appreciated by local consumers, particularly in the southern Iberian Peninsula. In the present study, headspace solid-phase microextraction coupled with gas chromatography-mass spectrometry (HS-SPME-GC/MS) was used to identify and quantify volatile organic compounds (VOCs), establishing a volatile fingerprint of dry-cured tuna throughout the manufacturing process. Unsupervised (PCA) and supervised (PLS-DA and sPLS-DA) multivariate statistical methods were applied to visualise, group, and classify the samples. A total of fifty-four VOCs were identified across the four steps involved in processing the final product. The ML-PLS-DA model demonstrated excellent discrimination (R^2^ = 0.912, Q^2^ = 0.878, and Accuracy = 1) for the samples. Additionally, ML-sPLS-DA was conducted to screen various VOC metabolites in the samples after both the salting and salt-washing steps; the levels of eighteen VOCs changed significantly (VIP > 1; *p* < 0.05). These results provide a theoretical basis for determining flavour formation and quality control in the traditional dry-curing process of tuna.

## 1. Introduction

Yellowfin tuna (*Thunnus albacares*) holds a foremost position in the global fish market due to its exceptional profile of macronutrients (lipids, fatty acids, protein, and amino acids) and micronutrients (essential elements, vitamins, and minerals). As a pelagic migratory fish, this species is widely distributed in tropical, subtropical and temperate waters, predominantly in the Pacific, Atlantic, and Indian oceans [1]. Consequently, yellowfin tuna serves as a vital source of revenue for numerous countries, including Japan, the European Union, and the United States [2]. Raw tuna is regarded as a delicacy and a premium product with an exceptionally brief shelf life, usually just a few days at refrigerated temperature. The limited shelf life is influenced by factors encompassing both food safety and quality, including alterations in colour, texture, flavour, and bacterial proliferation. These key physicochemical attributes of fish have a direct impact on consumer acceptance, as consumers are becoming more and more aware of food quality and safety. A wide range of preservation methods have been extensively used, including chilling or refrigeration, freezing, curing, smoking, canning, and drying [3,4,5,6]. To address these concerns, the European Union has promoted the implementation of good manufacturing practices and the development of Hazard Analysis and Critical Control Points (HACCP).

Dry-curing is probably the oldest traditional method of fish preservation, involving the application of salt to fresh fish flesh. This process initiates dehydration, which effectively reduces microbial growth and enzymatic reactions that may adversely affect fish quality [7]. Initially utilised for enhancing the storage and preservation of fresh fish, dry curing methods have gained popularity among consumers due to their ability to enhance the flavour and nutritional value of foods [8]. The unique aroma of fish products primarily arises from microbial carbohydrate metabolism, the action of endogenous proteases and fat decomposition. In some cases, the tuna-based products prepared with this type of technique are regional specialities, such as mojama. Mojama, a dry-cured tuna product, stands out as one of the most iconic food items derived from traditional tuna processing along the southern Iberian Peninsula (particularly in Andalusia) and Portugal [9]. The special organoleptic characteristics and the significant importance of mojama in its production area led to the establishment of two Protected Geographical Indications (PGIs) under the European Union’s quality schemes for agricultural products and foodstuffs: ’Mojama de Barbate’ [10,11] and ’Mojama de Isla Cristina’ [12,13]. Strong unpleasant flavours are the most serious issue faced by the fish industry, leading to consumer dissatisfaction and a decrease in the market value of the product. Furthermore, the flavour of dry-cured fish products is crucial in enhancing their palatability. Research has shown that the distinctive flavour of dried salted fish comes from volatile flavour precursors, primarily free fatty acids from lipolysis and free amino groups from protein hydrolysis [14,15]. These lipid oxidation products, such as aldehydes, ketones, alcohols, esters, and hydrocarbons, are highly volatile and have lower odour thresholds than other compounds, making them the main contributors to flavour. Curing processes, such as salting and drying, significantly influence volatile organic compounds (VOCs) generation in processed fish products. These steps are essential in product manufacturing as they contribute to the development of the final characteristic flavour of the product by enhancing lipid oxidation [8,16,17].

The scope of this research was to evaluate the changes in the volatile organic fingerprint during the traditional process of dry-cured tuna by employing an HS-SPME-GC/MS technique and to identify potential VOC markers for the different steps of processing using multivariate chemometric analysis. This approach seeks to optimise process monitoring, ensure product quality, and establish key VOC indicators for standardizing and improving industrial dry-curing practices.

## 2. Materials and Methods

### 2.1. Sampling

A total of 48 samples were selected for this study. They were obtained by processing yellowfin tuna (*Thunnus albacares*) captured from the FAO 34 fishing area (eastern Atlantic Ocean). All of them belonged to the Andalusian company included in the Protected Geographical Indication (PGI) for “Mojama de Isla Cristina”. The samples were collected between September and December 2020. Representative samples of tuna flesh (500 g), all of which were subjected to the same processing conditions, were taken from each fish at every stage of the dry-cured tuna manufacturing process: the “ronqueo”, in which tunas are manually cut by specialised workers following a traditional process, as well as salting, salt-washing, and dry-curing (detailed in Section 2.2). Each tuna sample was labelled with a unique identifier for traceability throughout the entire production process until the final product. During transportation to the laboratory, the fresh tuna samples were stored in ice to prevent spoilage, while the dry-cured tuna samples were preserved as established for human consumption (vacuum-packed and refrigerated). Upon arrival at the laboratory, all the samples were stored at −20 °C to prevent any additional changes until analysis.

### 2.2. Mojama Production

**Figure 1:**
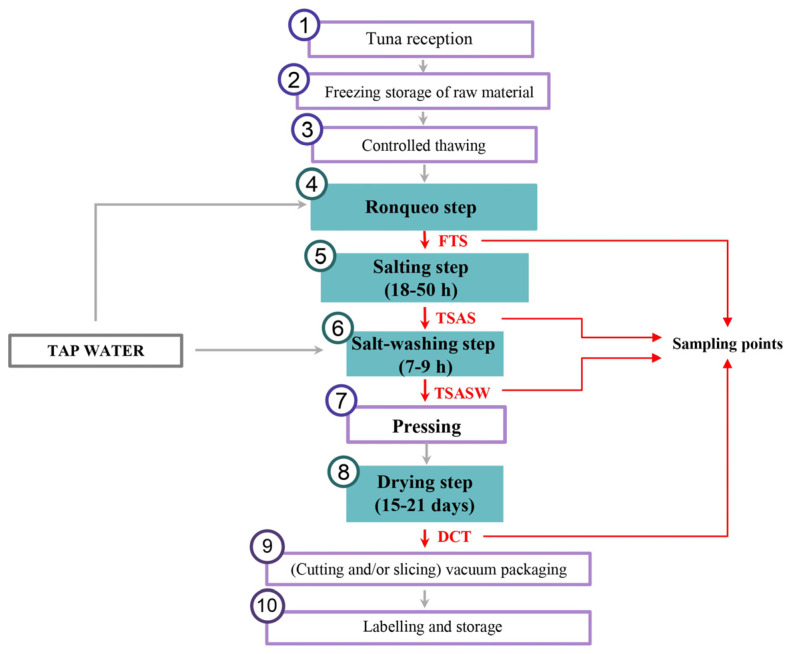
Process flow diagram for the traditional processing of dry-cured tuna steps: (1) tuna reception; (2) freezing storage of raw material; (3) controlled thawing; (4) Ronqueo (FTS: fresh tuna sample); (5) salting (TSAS: tuna sample after salting); (6) salt-washing (TSASW: tuna sample after salt-washing); (7) pressing; (8) drying (DCT: dry-cured tuna); (9) cutting and/or slicing, vacuum packaging; and (10) labelling and storage.

The traditional processing of dry-cured tuna (Figure 1) was carried out according to the Spanish Protected Geographical Indications (PGIs). The process begins with the decapitation and evisceration of the tuna. After washing with tap water, the tuna is cut into loin strips (≤5 cm) with the skin and bones removed in a manual process known as “ronqueo” (FTS: fresh tuna samples), performed by specialised operators. The ventral loins were selected for this study due to their higher fat content, which plays a crucial role in the development of flavour and aroma during the curing process. During salting, the tuna strips are coated in sea salt for 18 to 50 h to induce water and fat loss, thereby enhancing product stability (TSAS: tuna samples after salting). Subsequently, the salt is washed off in two phases: an initial rinse to remove surface salt and a subsequent immersion in cold water baths for 7–9 h to achieve the optimal salt concentration (TSASW: tuna samples after salt-washing). Next, the loin strips are pressed to facilitate further water and fat loss. Finally, the strips are dried horizontally for at least 2 days and then hung for 18 ± 3 days under controlled temperature (~16 °C) and humidity (~65%) conditions. The final product, dry-cured tuna (DCT), is vacuum-packed to maintain its physical and chemical properties, extending its shelf life to up to 6 months under refrigeration (5 ± 1 °C).

### 2.3. Chemicals and Reagents

Sodium chloride (NaCl, analytical grade) was obtained from Panreac (Barcelona, Spain). A standard alkane mixture ranging from C7 to C30 (≥97%) was purchased from Supelco (Bellefonte, PA, USA). Mili-Q water was purified using a Milli-Q Plus water system (Millipore, Spain). Analytical standards (97–99% purity) for various compounds, including hexanal, heptanal, octanal, nonanal, (Z)-4–Heptenal, (E)–2–octenal, (E,E)-2,4-heptadienal, (E)-2-nonenal, 4–ethyl–benzaldehyde, (E)-2–undecenal, (E)–2–dodecenal, benzaldehyde, benzeneacetaldehyde, (E,E)-2,4–decadienal, (E,Z)–2,6–nonadienal, (E,E)–2,4–Heptadienal, acetic acid, pentanoic acid, hexanoic acid, heptanoic acid, (Z)–2–penten–1–ol, 1–penten–3–ol, 2–ethyl–1–hexanol, heptylcyclohexane, (E)-3–hexen–1–ol, 1–octen–3–ol, 1–octanol, 1–hexanol, 1-pentanol, 1-heptanol, 2,7-Octadien-1-ol, 2,3–pentanedione, 2–nonanone, 2–undecanone, 2-pentylfuran, 2-ethylfuran, ethylbenzene, 1,3-dimethyl-benzene, p-xylene, styrene, trimethylpyrazine, tetradecane, tridecane, dodecane, pentadecane, and cyclohexene (D-limonene), were sourced from Sigma–Aldrich (Steinheim, Germany).

### 2.4. HS-SPME-GC/MS Analysis of VOCs

#### 2.4.1. Sample Preparation and HS-SPME-GC/MS Analysis

All the samples were manually ground for 30 s using a homogeniser (Sammic, Madrid, Spain). For each sample, one gram of the ground material and 3 mL of a 20% salt solution were placed into a 10 mL headspace vial. The mixture was homogenised with a vortex shaker for 30 s and placed in a Combi-Pal autosampler tray (CTC Analytics, Zwingen, Switzerland). A 50/30 µm DVB/CAR/PDMS fibre (Supelco) was used for the HS extraction according to previous experience [18]. During the equilibrium process, the vials were incubated at 500 rpm for 5 min at 50 °C, while the extraction was performed under the same agitation at 40 °C for 30 min. Analyses were performed in a Trace GC ultra gas chromatograph coupled to an ISQ Single MS spectrometer (Thermo Fisher Scientific, San Jose, CA, USA). Each injection was performed in splitless mode for 0.75 min, and chromatographic separation was achieved using an HP-FFAP column (50 m × 0.32 mm × 0.50 µm) (Agilent, Santa Clara, CA, USA). Helium (purity 99.99%) was used as the carrier gas at a flow rate of 1.7 mL/min. The oven temperature program started at 40 °C for 3 min, increased to 200 °C at 6 °C/min, and was maintained at this temperature for 15 min. The transfer line and ion source temperature were set at 230 °C and 200 °C, respectively. The mass spectra (MS) operated in electron ionization (EI) mode at 70 eV in the range of 50 to 450 *m*/*z* and the scanning frequency was 0.1 scans/s. The analyses were performed in duplicate for all samples.

#### 2.4.2. Data Processing and Identification of VOCs

Raw chromatograms were converted into .CDF format using the OpenChrom open-source software (OpenChrom^®^ 1.2.0 “Alder”, https://www.openchrom.net (accessed on 12 September 2024). Building upon prior work in our laboratory [19], deconvolution of the data was conducted employing a PARAllel FACtor analysis2 (PARAFAC2) model due to its capability to deconvolute co-eluted, low signal-to-noise ratios, and shifts in retention times with minimal hyperparameters required to be set [20,21]. First, the chromatograms were segmented into 145 intervals, ensuring that each peak was entirely contained within an interval for the entire set of chromatograms processed simultaneously. Each interval encompassed a maximum of 8 compounds, allowing the PARAFAC2 model to resolve the underlying and overlapping compounds in each interval [21]. Then, to assess each model, the core consistency and the fit were optimised, aiming to achieve values as close to 100% as possible for both parameters. Subsequently, all deconvoluted peaks underwent manual inspection. The mass spectrum resolved by PARAFAC2 was cross-referenced with the NIST database (version 2.0, NIST, Gaithersburg, MD, USA) for potential identification. The freely available Deconvolution and Identification System (PARADISe v.6.0.1) software (https://ucphchemometrics.com/paradise/, accessed on 12 September 2024) was utilised for this deconvolution procedure. Different levels of identification confidence were performed following the proposed minimum reporting criteria defined by the Metabolomic Standard Initiative [22]. Compounds were definitively annotated (level 1) by matching both the PARAFAC2 MS spectra and linear retention index (LRI) with purchased available standards. LRIs were computed using a mixture of standard alkanes (C7–C30) under identical conditions, according to the theory by van Den Dool and Kratz. Tentative identifications (levels 2 and 3) were established by comparing the MS spectra and LRIs with those in the National Institute of Standards and Technology (NIST02). Level 2 identification was assigned to compounds exhibiting a match factor (MF) > 850 and LRI ± 30 compared to the NIST database, while level 3 was assigned to compounds with an MF > 650 and LRIs ± 30 compared to the NIST database. To ensure the reliability of our results, a pooled quality control (QC) sample was repeatedly analysed throughout the experimentation process. This was done to mitigate any potential inaccuracies from compound degradation, variations in equipment settings, and/or sample preparation. Compounds exhibiting consistent and stable responses (with a relative standard deviation, RSD, of ≤20% in the QCs) were exclusively included in the method.

### 2.5. Data Analysis and Chemometrics

The normalised data were exported to R free software v.4.0.3 for statistical analysis. To examine differences in the dataset related to the factor under study (processing), a one-way analysis of variance (ANOVA) was conducted, followed by a post hoc Tukey’s honest significance differences (HSD) test at a significance level of 0.05. Venn diagrams were created using an online tool (https://bioinformatics.psb.ugent.be/webtools/Venn/, accessed on 12 September 2024). Subsequently, classification models were carried out with MATLAB 2017bR software (The Mathworks Inc., Natick, MA, USA), and the chemometrics tool Solo, running PLS_Toolbox (v.8.7). A multi-level principal component analysis (ML-PCA) was conducted to reduce data dimensionality, facilitating data exploration, visualization of potential trends and the detection of possible outliers. Following this, a multi-level partial least squares discriminant analysis (ML-PLS-DA) was applied to further distinguish the samples during the dry-cured tuna process. A permutation test (100 iterations) was performed to validate the ML-PLS-DA results and prevent overfitting [23]. Subsequently, the variable importance in projection (VIP) was used to identify the VOC markers for each processing step [24] (Chong and Jun, 2005). The multi-level sparse partial least squares–discriminant analysis (ML-sPLS-DA) approach was employed to partition the total variance into individual variations, enabling the identification of the specific effects on the response. Finally, MetaboAnalyst 6.0 (https://www.metaboanalyst.ca, accessed on 12 September 2024) software was utilised to conduct a volcano plot statistical analysis. This analysis combined fold change results and *p*-values from a *t*-test to visually highlight significant features based on their statistical significance, facilitating the selection of potential biomarkers for the two groups under comparison. This representation used the autoscaled intensity of all VOCs detected after HS-SPME-GC/MS analysis.

## 3. Results and Discussion

### 3.1. Volatile Organic Compounds Fingerprint

**Figure 2:**
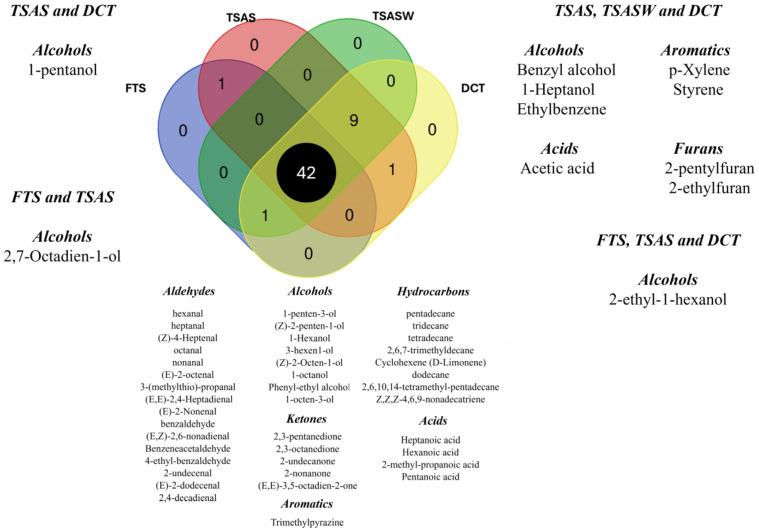
Venn diagram of volatile compounds present in fresh tuna samples (FTS), tuna samples after salting (TSAS), tuna samples after salt-washing (TSASW), and dry-cured tuna (DCT).

As shown in Table 1, a total of 54 VOCs were identified in the samples across the different steps of the manufacturing process of dry-cured tuna. The 54 VOCs were classified into seven chemical classes, comprising aldehydes (16), alcohols (13), ketones (5), acids (5), aromatics hydrocarbons (5), aliphatic hydrocarbons (8), and furans (2). Significant differences in the identified VOCs were observed among the different stages of the traditional manufacturing process, providing information about the quantitative changes. In our study, a Venn diagram (Figure 2) was used as a quick and visual tool to display the volatile fingerprint throughout the dry-cured tuna manufacturing process. In summary, the FTS exhibited the simplest volatile profile in terms of the number of identified compounds, since they had not undergone any processing that would develop different flavour and aroma precursors.

These samples presented a total of 44 compounds. Specifically, 42 of these compounds, including aldehydes, alcohols, ketones, hydrocarbons, acids, and one aromatic VOC, were present in every processing step. The most notable trend was the formation of certain alcohols, furans, and aromatic hydrocarbons after the salting step. Finally, the number of volatiles increased to 53 compounds during the dry-cured tuna manufacturing process. The findings of this study have significant practical applications in the food industry, particularly in the production and regulation of traditional dry-cured tuna. The identification of key VOCs throughout the curing process provides a scientific basis for improving flavour consistency in production. Previous studies, such as Wan et al. [25], have demonstrated that monitoring volatile profiles enables manufacturers to optimise curing time and storage, ensuring consistent sensory quality. Similarly, Leni et al. [26] highlighted the use of VOCs and other biomarkers to authenticate and monitor the quality of long-ripened products with Protected Designation of Origin (PDO). These methods can also be applied to other traditional foods, ensuring quality and compliance with regulatory standards.

#### 3.1.1. Aldehydes

In our study, the total aldehydes content increased from 1.23 mg/kg in the fresh tuna to 3.66 mg/kg during the salting step, followed by a decline to 2.10 mg/kg in the samples obtained during the salt-washing step and a subsequent increase in DCT (3.05 mg/kg) (Table 1). This increase after salting could be attributed to the oxidation of lipids, a key step in traditional dry-cured tuna production (Figure 1), which has been observed in other dry-cured fish products [17,27,28]. Moreover, salting leads to water loss in fish flesh, resulting in a concentration effect on compounds within the matrix, including VOCs. The decrease in aldehydes between salting and salt-washing could be explained by the dilution effect of water added during rinsing. Aldehydes are products of lipid oxidation, catalysed by endogenous enzymes and microorganisms [29]. They have already been pinpointed as the predominant VOCs in dry-cured fish, playing a pivotal role in the development of the distinctive flavour profile of these products. Aldehydes impart a characteristic fish-like odour, with low olfactory thresholds [30].

Of the total aldehydes detected, approximately 60% comprised saturated (hexanal, heptanal, etc.) and unsaturated ((Z)-4-heptenal, (E)-2-octenal, etc.) linear aldehydes (Table 1). Linear aldehydes within the 6-to-10 carbon atom range are widely recognised as secondary products from the oxidation and enzymatic breakdown of polyunsaturated fatty acids (PUFAs) [30,31]. These compounds have been previously identified as significant volatile flavour components in various dry-cured fish products [27,32,33,34]. Hexanal (notes: grass, tallow, fat), heptanal (notes: fat, citrus, rancid), octanal (notes: fat, soap, lemon, green), and nonanal (notes: fat, citrus, green) were found at all stages of dry-cured tuna production. These compounds, originating from the oxidation of linoleic acid (hexanal) and oleic acid (heptanal, octanal, and nonanal), displayed a significant increase from FTS to those obtained in the salting step. Specifically, hexanal, heptanal, octanal, and nonanal exhibited a remarkable three-fold increase (*p* < 0.001) from fresh tuna to the salting step (46.98 vs. 113.96, 22.19 vs. 56.98, 8.96 vs. 34.46, and 91.56 vs. 210.67 µg/kg, respectively). This supports hexanal as a reliable indicator of lipid oxidation, amplified by the salting process [17].

The unsaturated aldehydes (Z)-4-heptenal and (E)-2-octenal exhibited a similar trend, demonstrating a significant increase (*p* < 0.001, *p* < 0.05) between FTS and both the samples from the different processing steps. (Z)-4-heptenal (notes: biscuit, creamy, dairy, fatty, green, milky, and oily) and (E)-2-octenal (notes: banana, cucumber, fat, fresh, green, herbal, leaf, nut, and waxy) have been identified in the literature [35,36] as products of the enzymatic breakdown of lipids in fish, likely originating from the oxidation of α-linolenic and linoleic acid, respectively. Additionally, 3-(methylthio)-propanal, a sulphur-containing compound previously recognised in salted and dry-cured fish products [8], increased from 163 µg/kg in FTS to 1882 µg/kg after the salting stage. However, the concentration of this aldehyde decreased to 1511 µg/kg after TSAS and DCT. Previously, Wang et al. [37] demonstrated that this VOC, which imparts a roasted potato aroma, can be synthesised from methionine via the Strecker degradation pathway and further microbial-driven degradation.

Lastly, three cycloaldehydes were identified: benzaldehyde (notes: almond, bitter, burnt sugar, cherry, sharp, strong, and sweet), benzeneacetaldehyde (notes: floral and fruity) and 4-ethyl-benzaldehyde (notes: almond, bitter, and sweet). Particularly, the latter two emerged as the most abundant aldehydes throughout the entire manufacturing process, initially detected at concentrations of 364 µg/kg and 253.10 µg/kg, respectively, in FTS. They increased significantly during the salting step, reaching concentrations of 546 µg/kg and 445.43 µg/kg, respectively, following a similar pattern to that observed for the linear aldehydes (both saturated and unsaturated) discussed above. Even aromatic aldehydes, products from the degradation of amino acids, were previously identified in salted and dry-cured fish products [27,28,34,38].

#### 3.1.2. Alcohols

The total concentration of alcohol family compounds significantly increased with processing, from the fresh tuna (11.08 µg/kg) to the dry-cured tuna product (158.2 µg/kg). This increase may be attributed to lipid oxidation. Alcohols are VOCs primarily formed through the oxidation of PUFAs and the reduction of sugars, amino acids, and carbonyl compounds in fish tissues [39], and contribute significantly to the aroma of dry-cured fish [40].

In our study, 1-penten-3-ol was the compound with the highest contribution in FTS, representing 67% of this family (Table 1). Throughout the manufacturing process, this VOC increased to 12.75 µg/kg after dry-curing. Interestingly, this increase in the concentration within the matrix could be attributed to water loss following the salting and drying of the tuna loins, as well as to enhanced lipid oxidation during the curing process [34]. The compound 1-penten-3-ol is primarily formed through the action of lipoxygenases on long-chain n-3 and n-6 PUFAs, but can also result from microbial spoilage activity, which leads to the degradation of amino acids and lipids [41,42]. It is recognised for presenting various odours, aromas and flavours, including burnt, meaty, and pungent odours [43]; fishy and grassy aromas [44]; fatty, hay-like, or grassy flavours [45]; as well as solvent, vegetal, and spicy aroma [46].

On the other hand, the concentration of 1-octen-3-ol in the fresh tuna nearly doubled after the salting step (0.48 vs. 0.88 µg/kg). This increase is likely due to the oxidation of linoleic and arachidonic acid caused by the action of salt during the salting phase in the manufacturing process of dry-cured tuna [47]. This VOC has been previously described as a characteristic volatile compound in dry-cured fish, such as cod [48] and mackerel [32]. It is known for its fermented mushroom odour [49], and has also been described as possessing a fresh fish aroma [50], as well as earthy, herbaceous, and spicy aromas [51]. Both compounds, 1-penten-3-ol and 1-octen-3-ol, have been reported to be useful quality markers for fish products [39,52].

Interestingly, neither 1-pentanol nor 1-heptanol were initially detected in the fresh tuna, but both emerged during the salting stage. It is noteworthy that the increase in alcohol concentration during processing is due to the appearance of 1-heptanol after the salting process, reaching values of 139.11 µg/kg in the final product. This compound is derived from the auto-oxidation of n-3 and n-6 PUFAs [39].

Lastly, two-branched alcohols, namely phenyl-ethyl alcohol and 2-ethyl-1-hexanol, were detected during the processing of dry-cured tuna (Table 1), produced through the Strecker degradation of amino acids [53].

#### 3.1.3. Ketones

Ketones constitute another class of VOCs whose presence in dry-cured fish products has been linked to both lipid oxidation and microbial activity [27,28]. However, it has been suggested that ketones have a higher odour threshold compared to aldehydes and alcohols, thereby their contribution to the development of typical or off flavours can be considered less crucial [54]. In this study, five ketones were identified in the samples analysed during the manufacturing process of mojama, with significant fluctuations. Generally, the total concentration of ketones significantly increased from FTS (179.71 µg/kg) to the TSAS (246.60 µg/kg) and TSASW (260.05 µg/kg), followed by a significant decrease in the samples of DCT (179.87 µg/kg).

More than 80% of the total ketones detected throughout the entire manufacturing process of dry-cured tuna were accounted for by 2,3-octanedione and (E,E)-3,5-octadien-2-one, both described as characteristic VOCs in dry-cured fish [28] and meat [54,55] products. Particularly, (E,E)-3,5-octadien-2-one has been classified among the VOCs associated with the formation of the processing odour in fish products [56]. In our study, (E,E)-3,5-octadien-2-one was detected at the lowest concentration during the first steps of processing (41.72 µg/kg in FTS and 68.61 µg/kg and those obtained after salting), having the highest concentration (*p* < 0.05) in the samples obtained after the salt-washing (74.89 µg/kg) and drying steps (96.97 µg/kg). Our findings are in line with those reported by Moretti et al. [27]. However, no ketones typically associated with microbial spoilage, such as acetoin or methyl ketones were detected at any stage of mojama processing in our study [14,34,57]. This suggests that the traditional manufacturing process used to transform fresh tuna loins into dry-cured tuna, as examined in this study, adheres to the best production practices.

#### 3.1.4. Furans

In our study, two furan compounds, 2-ethylfuran and 2-pentylfuran, were identified. Both compounds were generated during the processing of dry-cured tuna and increased significantly, from 27.98 µg/kg after the salting step to 60.66 µg/kg in the dry-cured tuna (Table 1). These furans were not identified in FTS. Furans are usually generated via pathways involving Amadori rearrangement or in dehydrated carbohydrate condensates [43]. Furthermore, furans and their derivatives can also result from the oxidation of fatty acids [58].

The compound 2-ethylfuran, representing 73% of the total furan family in TSAS, tended to double in concentration when the tuna was dry-cured, where this VOC constituted 83% of the total furans. The formation of this compound can be explained by considering that the 12-hydroperoxide of α-linolenic acid, the 14-hydroperoxide of EPA (20:5n-3), and the 16-hydroperoxide of DHA (22:6n-3) can undergo β-cleavage to generate a conjugated diene radical. This radical can subsequently react with oxygen to form a vinyl hydroperoxide. Cleavage of the vinyl hydroperoxide, triggered by the removal of a hydroxyl radical, leads to the formation of an alkoxyl radical. This radical then undergoes cyclization, ultimately resulting in the production of 2-ethylfuran [59]. Similarly, 2-pentylfuran, a well-known autoxidation product of linoleic acid, increased significantly from 7.47 to 10.01 µg/kg in the final product (Table 1). Furans play a significant role in enhancing the aroma of fish and meat products due to their low odour threshold values. Both 2-ethylfuran and 2-pentylfuran contribute to a pleasant aroma profile [60] with sweet, green, fruity, vegetable aromatic notes and roasted nuances [61,62].

#### 3.1.5. Acids

In this study, five organic acids were identified. On average, the concentration of total acids increased significantly during processing (*p* < 0.001), ranging from 0.82 µg/kg in FTS to 1.24–1.56 µg/kg in TSAS and TSASW, and reaching 2.26 µg/kg in DCT (Table 1).

Two VOCs in this category followed a similar trend. 2-methyl propanoic acid (with rancid, buttery, cheesy, and hammy notes [63]) was detected at its highest concentration in the dried product (0.48 µg/kg). The formation and concentration increase in this VOC could potentially stem from the catabolism of amino acids, particularly L-valine [64]. Similarly, acetic acid was not found in FTS as expected, but it appeared in similar concentrations in samples from the salting and salt-washing steps (0.12 µg/kg), reaching the highest concentration (0.20 µg/kg) in DCT. Acetic acid is an important odour-active compound imparting vinegar-like, cheesy, sweaty and sour aromas [65]. In the literature, it has been described in dry-cured meat products such as ham, although its exact origin remains unclear [66]. One possible pathway for acetic acid formation is through the Maillard reaction [67]. However, VOCs like pentanoic acid and heptanoic acid showed an increase in concentration in samples after the salting step (1.09 and 0.19 µg/kg, respectively) and decreased after the salt-washing step, reaching concentrations similar to those in FTS.

#### 3.1.6. Aliphatic Hydrocarbons

Additionally, several aliphatic hydrocarbons were detected during the manufacturing process of dry-cured tuna. These compounds are thought to originate from aquatic sources. Specifically, they can be generated by microorganisms and cyanobacteria and may enter the fish through environmental contamination, being absorbed directly from water [68,69]. The total hydrocarbon content increased gradually from 98.59 μg/kg in the fresh tuna loins to 164.58 μg/kg during salting, then decreased to 123.14 μg/kg during washing, and finally to 102.51 μg/kg in mojama (Table 1). Hydrocarbons are generally formed through lipid oxidation, and their presence in mojama could be attributed to the product’s dehydration and oxidation induced by the salting and drying process. Although many hydrocarbons have been associated with the alkoxy radical homolysis of fatty acids, they do not generally make a significant contribution to the flavour of foodstuffs, including fish products, due to their high odour threshold levels [70,71]. Therefore, the differences in the concentration of hydrocarbons detected in our study can be considered negligible.

#### 3.1.7. Aromatic Hydrocarbons

Finally, among the compounds identified in this group, we found the following: ethyl benzene, 1,3-dimethyl-benzene, p-xylene, trimethyl pyrazine and styrene. The total amount of aromatic hydrocarbons significantly increased from the samples of fresh tuna (0.12 μg/kg) to the samples obtained after the salting and washing (13.42 μg/kg and 14.64 μg/kg, respectively) stages, followed by a noticeable decrease in the samples of dry-cured tuna (5.72 μg/kg). The major contributor to the total concentration was the ethylbenzene. Most of these compounds have previously been reported in different kinds of fish such as dried scallops (*Patinopecten yessoensis*) [72], and wild gilthead sea bream (*Sparus aurata*) [52,73]. However, the presence of these compounds in fish products can be attributed to multiple factors, including bioaccumulation from the aquatic environment, dietary intake, and potential formation during processing. While environmental contamination is a well-documented source of polycyclic aromatic hydrocarbons (PAHs) in marine organisms, certain processing methods, such as smoking, drying, and high-temperature treatments, can also contribute to their formation. Therefore, understanding the relative contribution of each source remains a subject of ongoing research [33,74].

### 3.2. Multivariate Statistical Analysis

**Figure 3:**
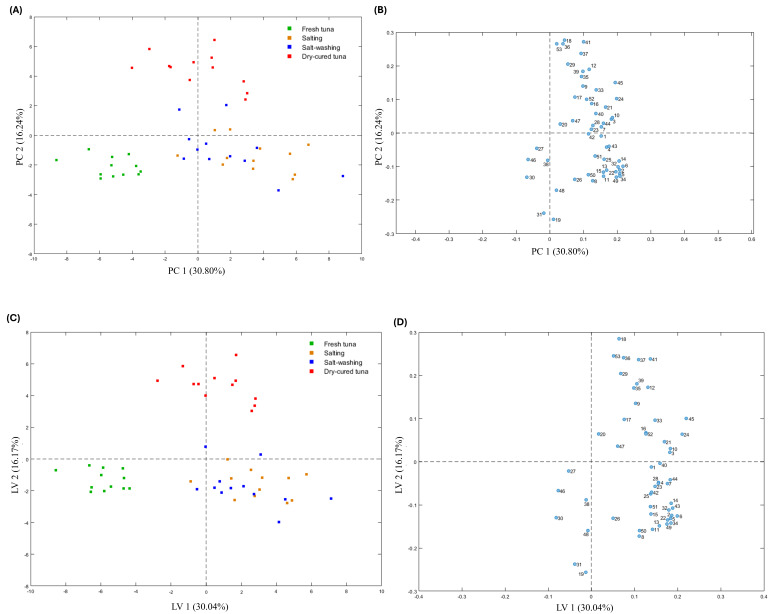
(**A**) Score plot of processing dry-cured tuna samples analysed in the study obtained by a multilevel principal component analysis (ML-PCA), (**B**) Loading plot of parameters measured in analysed samples obtained by ML-PCA. (**C**) Score plot of processing dry-cured tuna samples analysed in the study obtained by a multi-level Partial Least Squares Discriminant Analysis (ML-PLSDA), (**D**) Loading plot of parameters measured in analysed samples obtained by ML-PLSDA. Variables associated with higher loadings on the 1st or the 2nd principal component (PC1 and PC2 on the x and the y axis, respectively) are related to a higher influence on variability recorded among samples in the multi-level data matrix. 1 = hexanal; 2 = heptanal; 3 = (Z)-4-heptenal; 4 = octanal; 5 = nonanal; 6 = (E)-2-octenal; 7 = 3-(methylthio)-propanal; 8 = (E,E)-2,4-heptadienal; 9 = (E)-2-nonenal; 10 = benzaldehyde; 11 = (E,Z)-2,6-nonadienal; 12 = benzeneacetaldehyde; 13 = 4-ethyl-benzaldehyde; 14 = 2-undecenal; 15 = (E)-2-dodecenal; 16 = 2,4-decadienal; 17 = 1-penten-3-ol; 18 = 1-pentanol; 19 = (Z)-2-penten-1-ol; 20 = 1-hexanol; 21 = 3-hexen-1-ol; 22 = 1-octen-3-ol; 23 = 1-heptanol; 24 = 2-ethyl-1-hexanol; 25 = 1-octanol; 26 = (Z)-2-octen-1-ol; 27 = 2,7-octadien-1-ol; 28 = benzyl alcohol; 29 = phenyl-ethyl alcohol; 30 = 2,3-pentanedione; 31 = 2,3-octanedione; 32 = 2-nonanone; 33 = (E,E)-3,5-octadien-2-one; 34 = 2-undecanone; 35 = Acetic acid; 36 = 2-methyl-propanoic acid; 37 = Pentanoic acid; 38 = Hexanoic acid; 39 = Heptanoic acid; 40 = 2-ethylfuran; 41 = 2-pentylfuran; 42 = Ethylbenzene; 43 = 1,3-dimethyl-benzene; 44 = P-xylene; 45 = Styrene; 46 = trimethylpyrazine; 47 = 2,6,7-trimethyldecane; 48 = dodecane; 49 = Cyclohexene (D-Limonene); 50 = tridecane; 51 = tetradecane; 52 = pentadecane; 53 = 2,6,10,14-tetramethyl-pentadecane; 54 = Z,Z,Z-4,6,9-nonadecatriene.

Volatilomics, a subfield of metabolomics, focuses on detecting, characterizing and quantifying all volatile metabolites within a biological system. This discipline enables the accurate comparison of volatile compound profiles among sample groups and facilitates the identification of discriminatory compounds. The significance of volatilomics extends across various food areas, including food safety, food quality and food authenticity [75]. To ensure comparability, it is essential to normalise the data before proceeding with its analysis [76]. As part of an unbiased data exploratory analysis, all the volatile organic compounds (VOCs) underwent multi-level principal component analysis (ML-PCA). ML-PCA is an unsupervised chemometric method used to reduce the complexity of data and visually represent the primary correlations and variability within a dataset [69]. Figure 3A,B shows the scores plot of the first two components (PC1 and PC2), which collectively accounted for 30.80% and 16.24% of the variance, respectively. The plot revealed the formation of three distinct clusters: FTS (depicted by green squares), TSAS and TSASW (depicted by blue and orange squares, respectively), and DCT (depicted by red squares). In particular, an inherent separation is noticeable between the green and red group of samples, situated on opposing sides of the positive and negative halves of PC2, suggesting distinct volatile compound fingerprints. To understand the specific differences and to identify biomarkers associated with each of the four distinct steps, further clustering was required to form four groups corresponding to the manufacturing process. Thus, to obtain a more comprehensive understanding of the dissimilarities among the samples, an ML-PLS-DA model was developed with a confidence interval of 95%. The results of ML-PLS-DA were similar to ML-PCA (Figure 3C,D).

**Figure 4:**
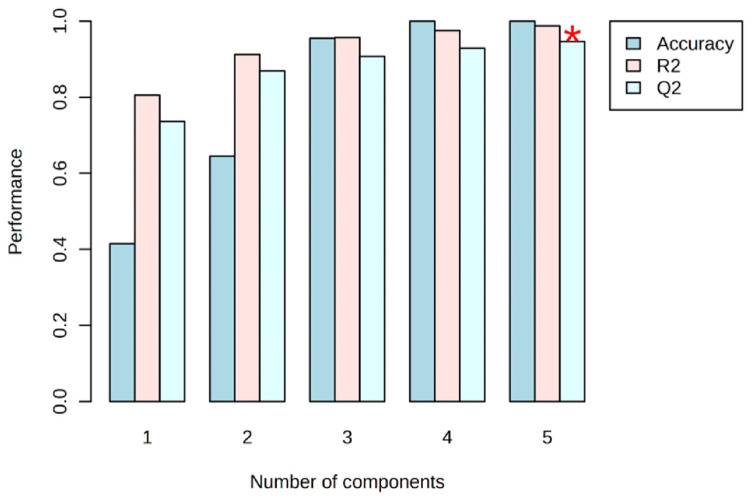
PLS-DA classification using different number of components. The red star (*) indicates the best classifier. R^2^ and Q^2^, greater than 0.5 indicates a model with reasonable fit with good predictive power.

The scores plot of ML-PLS-DA showed 30.04% and 16.17% variances by component 1 and component 2, respectively. Data points from the samples obtained after the salting and salt-washing processes overlapped, indicating that there was little change in some volatile substances in tuna flesh during the two production steps. Meanwhile, data points belonging to the FTS and DCT samples had no crossover and a long distribution distance, suggesting that the VOC fingerprints of both groups of samples were significantly different. This fact could be explained by the tentative transformation/degradation of proteins, fats, and other substances in FTS and, therefore, the production of volatile substances by protein hydrolysis, lipid oxidation, and glycolysis [18]. To test the proper fitting and robustness of the model, a 10-fold cross-validation method was used to indicate its sensitivity and predictive ability. Although the optimal performance could be achieved with five components (Figure 4), satisfactory modelling and prediction results were obtained using the first two PCs. In our study, we achieved R^2^ = 0.912 and Q^2^ = 0.878, indicating a robust predictive capability. Further validation of the supervised model involved conducting 1000 permutation tests (Figure S1).

**Figure 5:**
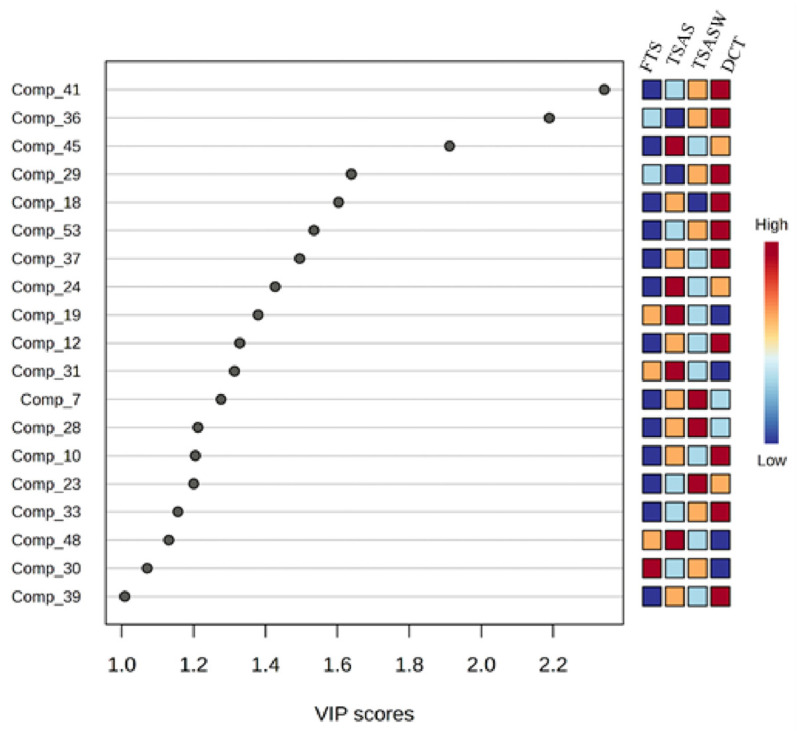
Important volatiles (VIP > 1.0) identified by PLS-DA. The coloured boxes on the right indicate the relative concentrations of the corresponding volatiles at different stages. 41 = 2-pentylfuran; 36 = 2-methyl-propanoic acid; 45 = styrene; 29 = phenyl-ethyl alcohol; 18 = 1-pentanol; 53 = Z,Z,Z-4,6,9-nonadecatriene; 37 = pentanoic acid; 24 = 2-ethyl-1-hexanol; 19 = (Z)-2-penten-1-ol; 12 = benzeneacetaldehyde; 31 = 2,3-octanedione; 7 = 3-(methylthio)-propanal; 28 = benzyl alcohol; 10 = benzaldehyde; 23 = 1-heptanol; 33 = (E,E)-3,5-octadien-2-one; 48 = cyclohexene (D-Limonene); 30 = 2,3-pentanedione; 39 = heptanoic acid.

The resulting distributions confirmed the model’s significant ability to predict VOCs in the sample groups, with a *p*-value < 0.001. VIP values were obtained for all 54 features used in developing the ML-PLS-DA model. Subsequently, several feature reductions were applied based on the criteria where variables with scores < 1 were discarded and scores ≥ 1 and *p* < 0.05 were considered as potential key markers for classification. The higher the VIP value, the greater the difference between groups, and the more important it is for discriminant classification [77]. Accordingly, heptanoic acid (Comp_39), 2,3-pentanedione (Comp_30), D-Limonene (Comp_48), (E,E)-3,5-octadien-2-one (Comp_33), 1-heptanol (Comp_23), benzaldehyde (Comp_10), benzyl alcohol (Comp_28), 3-(methylthio)-propanal (Comp_7), 2,3-octanedione (Comp_31), benzeneacetaldehyde (Comp_12), (Z)-2-penten-1-ol) (Comp_19), 2-ethyl-1-hexanol (Comp_24), pentanoic acid (Comp_37), Z,Z,Z-4,6,9-nonadecatriene (Comp_53), 1-pentanol (Comp_18), phenyl-ethyl alcohol (Comp_29), styrene (Comp_45), 2-methyl-propanoic acid (Comp_36) and 2-pentylfuran (Comp_41) were selected as the most important markers responsible for the discrimination between the different groups of samples (Figure 5). The VIP algorithm was used to minimise the number of features and to establish these 19 compounds as the most important features explaining the variation. Interestingly, the levels of two aldehydes (benzeneacetaldehyde, benzaldehyde), two alcohols (phenyl-ethyl alcohol, 1-pentanol), three acids (2-methyl-propanoic acid, pentanoic acid, heptanoic acid), 2-pentylfuran, Z,Z,Z-4,6,9-nonadecatriene and (E,E)-3,5-octadien-2-one were higher in the dry-cured tuna. Moreover, only 2,3-pentanedione was found in high levels in FTS. It has already been reported as an indicator of lipid oxidation in chilled fish muscle [33].

**Figure 6:**
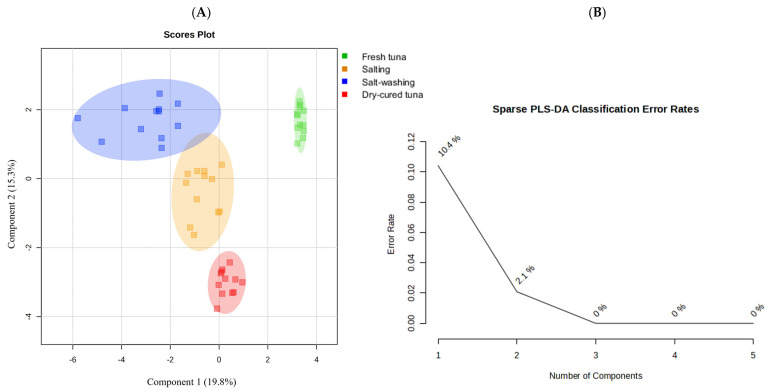
(**A**) ML-sPLS-DA score plots between selected PCs showing supervised clustering and separation between the four different steps during the manufacturing process of dry-cured tuna, (**B**) Classification error rates for each ML-sPLS-DA component.

The classification achieved through the PLS-DA statistical analysis could be enhanced further. Specifically, in the PLS-DA model, the estimation of the statistical parameters was carried out taking into account all the variables, even though some of them are not significant for the model [78]. The existence of non-significant variables poses a disruptive factor in the statistical framework, diminishing the model’s efficacy [79]. A typical procedure to reduce these variables and enhance the outcome of the statistical analysis involves applying a sparse approach to PLS-DA, which eliminates irrelevant variables during the calibration process [80,81]. In this sense, it was decided to carry out an ML-sPLS-DA approach to identify the individual effects on the response. Figure 6A and Figure 6B show the ML-sPLS-DA plot and the components chosen based on their classification error, respectively. As expected, the optimised statistical model made it possible to separate the samples obtained after the salting and salt-washing steps depending on their volatile composition. ML-sPLS-DA statistical analysis also enabled an only VOC fingerprint linked to each processing step to be determined.

**Figure 7:**
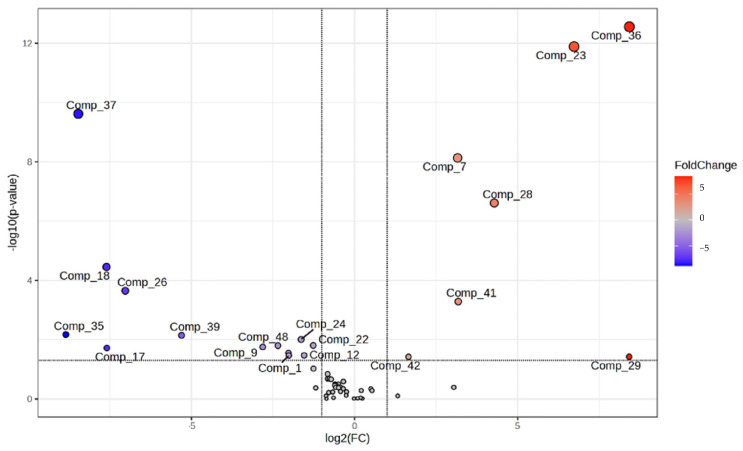
Volcano plot designed with the intensity of the initial 54 VOCs detected in the HS-SPME-GC/MS analysis of tuna samples. Coloured dots correspond to those VOCs with a higher intensity in tuna samples after the salt-washing (red) and salting (blue) steps. The numerical identification of the compounds can be observed in column 1 of Table 1.

Alongside the VIP evaluation in ML-sPLS-DA, the significance of some of these VOCs was confirmed by an additional statistical test, the volcano plot, which highlights the relevance of VOCs showing significant differences between samples after the salting and salt-washing steps. The volcano plot analysis used a fold-change (FC) of 2.0 and a false discovery rate of *p* < 0.05 [82] for all 54 VOCs initially identified. This type of representation made it possible to discern, after a quick observation, those VOCs that obtained the greatest changes and were also significant. Figure 7 shows that seven VOCs (red dots) were up-regulated (FC(TSASW/TSAS) > 1), thirteen volatile VOCs (blue dots) were down-regulated (FC(TSASW/TSAS) < 1), while the grey dots show thirty-three undifferentiated VOCs. This means that seven VOCs, numbered as 2-methyl-propanoic acid (Comp_36), 1-heptanol (Comp_23), 3-(methylthio)-propanal (Comp_7), benzyl alcohol (Comp_28), 2-pentylfuran (Comp_41), phenyl-ethyl alcohol (Comp_29) and Ethylbenzene (Comp_42), were significant for the group of the TSASW, while 13 features [pentanoic acid (Comp_37), 1-pentanol (Comp_18), (Z)-2-octen-1-ol (Comp_26), acetic acid (Comp_35), 1-penten-3-ol (Comp_17), 2-ethyl-1-hexanol (Comp_39), dodecane (Comp_48), 2-ethyl-1-hexanol (Comp_24), (E)-2-nonenal (Comp_9), 2-ethyl-1-hexanol (Comp_24), hexanal (Comp_1), 1-octen-3-ol (Comp_22), and benzeneacetaldehyde (Comp_12)] were associated with the TSAS. The VOCs numbered 2-methyl-propanoic acid (Comp_36), 1-heptanol (Comp_23), 3-(methylthio)-propanal (Comp_7), benzyl alcohol (Comp_28) and 2-pentylfuran (Comp_41) were the most significant in the TSASW samples, whereas the features pentanoic acid (Comp_37), 1-pentanol (Comp_18), (Z)-2-octen-1-ol (Comp_26), acetic acid (Comp_35), and heptanoic acid (Comp_39) were the VOCs with the highest significance in the TSAS group.

## 4. Conclusions

In this study, we used HS-SPME-GC/MS and multivariate statistical analysis to identify the dynamic changes that occur in the VOC fingerprint of dry-cured tuna during the manufacturing process. Fifty-four VOCs, including aldehydes, alcohols, ketones, acids, furans, aromatic hydrocarbons, and aliphatic hydrocarbons, were identified and quantified. PCA analysis of the VOCs showed that three clusters of samples (fresh tuna, dry-cured tuna, and those obtained after the salting and salt-washing steps) were significantly distinguished on the score map. To understand the specific differences and identify biomarkers associated with each of the four distinct processing steps, further clustering was required. In this sense, an ML-PLS-DA model was constructed based on the VIP values. The VIP algorithm was used to minimise the number of features and establish 19 compounds as the most important features explaining the greatest variation. In addition, ML-sPLS-DA and volcano plot approaches were applied to enable the identification of the individual effects on the response between the tuna samples after the salting and salt-washing steps, where seven VOCs were up-regulated and thirteen VOCs were down-regulated. The use of several advanced techniques to analyse volatile metabolites offered a thorough and detailed insight into the fingerprint of volatile organic compounds in the final product (dry-cured tuna), as well as its modifications and transformations along the different steps of the manufacturing process.

## Figures and Tables

**Table 1:** Volatile organic compounds fingerprint content (μg/kg) during the industrial processing of dry-cured tuna.

No		Identification ^a^	RT ^b^	LRI_exp_	LRI_lit_	Steps				
						FTS	TSAF	TSASW	DCT	*p*-Value
	*Aldehydes*									
1	hexanal	MS,STD,LRI	9.37	1102	1106	46.98 ± 27.22 c	113.96 ± 55.00 a	62.02 ± 34.19 bc	92.04 ± 77.03 ab	***
2	heptanal	MS,STD,LRI	11.92	1203	1208	22.19 ± 14.00 b	56.98 ± 29.20 a	55.03 ± 47.03 a	35.07 ± 27.48 ab	***
3	(Z)-4-heptenal	MS,STD,LRI	13.47	1267	1255	3.81 ± 1.85 b	10.10 ± 2.33 a	8.83 ± 5.67 a	9.49 ± 4.90 a	***
4	octanal	MS,STD,LRI	14.47	1308	1314	8.96 ± 7.00 b	34.46 ± 22.53 a	36.12 ± 24.86 a	20.12 ± 11.70 ab	**
5	nonanal	MS,STD,LRI	16.95	1415	1406	91.56 ± 42.98 b	210.67 ± 106.24 a	218.27 ± 117.64 a	132.78 ± 74.61 ab	**
6	(E)-2-octenal	MS,STD,LRI	17.89	1458	1436	0.02 ± 0.00 b	0.03 ± 0.01 a	0.03 ± 0.01 a	0.03 ± 0.01 a	*
7	3-(methylthio)-propanal ^1^	MS,LRI	18.86	1490	1480	163 ± 26.90 d	1882 ± 419.23 a	1521 ± 522.78 b	1511 ± 323.12 c	***
8	(E,E)-2,4-heptadienal	MS,STD,LRI	19.40	1528	1512	182.53 ± 64.36	228.66 ± 106.38	227.21 ± 115.59	175.90 ± 92.74	ns
9	(E)-2-nonenal	MS,STD,LRI	20.15	1564	1542	57 ± 15.02 b	88 ± 35.74 a	59 ± 19.42 b	85 ± 37.43 a	***
10	benzaldehyde	MS,STD,LRI	20.19	1566	1556	0.12 ± 0.07 b	0.27 ± 0.08 a	0.24 ± 0.07 a	0.26 ± 0.09 a	**
11	(E,Z)-2,6-nonadienal	MS,STD,LRI	21.27	1618	1629	0.84 ± 0.81	1.05 ± 0.86	1.56 ± 1.0	0.96 ± 0.68	ns
12	Benzeneacetaldehyde	MS,STD,LRI	22.61	1685	1678	364 ± 90.90 b	546 ± 186.40 a	437 ± 187.00 b	629 ± 184.09 a	***
13	4-ethyl-benzaldehyde	MS,STD,LRI	23.90	1752	1730	253.10 ± 90.81 b	445.43 ± 186.40 a	438.79 ± 187.00 a	310.46 ± 184.10 b	**
14	(E)-2-undecenal	MS,STD,LRI	24.45	1781	1736	0.40 ± 0.17 b	1.37 ± 0.99 a	1.33 ± 1.57 a	0.91 ± 0.56 ab	*
15	(E)-2-dodecenal	MS,STD,LRI	24.89	1804	1835	0.80 ± 0.26 c	1.35 ± 0.95 a	1.34 ± 0.71 a	0.98 ± 0.63 b	*
16	(E,E)-2,4-decadienal	MS,STD,LRI	25.66	1824	1824	35.61 ± 15.34	40.58 ± 12.23	39.58 ± 10.83	40.52 ± 22.95	ns
	Sum of aldehydes ^c^					1.23 ± 0.385 c	3.66 ± 1.20 a	2.10 ± 0.33 b	3.05 ± 1.04 a	**
	*Alcohols*									
17	1-penten-3-ol	MS,STD,LRI	11.27	1177	1192	7.42 ± 3.81 b	10.10 ± 4.48 ab	7.10 ± 3.54 b	12.75 ± 8.15 a	**
18	1-pentanol	MS,STD,LRI	12.52	1228	1241	-	1.004 ± 0.70 b	-	2.42 ± 0.52 a	***
19	(Z)-2-penten-1-ol	MS,STD,LRI	15.20	1339	1325	2.02 ± 1.23 ab	2.10 ± 2.91 ab	3.12 ± 2.27 a	0.69 ± 0.55 b	***
20	1-hexanol	MS,STD,LRI	15.88	1573	1368	0.47 ± 0.20	0.48 ± 0.22	0.46 ± 0.32	0.56 ± 0.42	ns
21	(E)-3-hexen-1-ol	MS,STD,LRI	16.67	1403	1384	0.047 ± 0.004	0.053 ± 0.004	0.053 ± 0.003	0.053 ± 0.005	ns
22	1-octen-3-ol	MS,STD,LRI	18.03	1464	1438	0.48 ± 0.20 b	0.88 ± 0.21 a	0.66 ± 0.18 b	0.61 ± 0.38 b	*
23	1-heptanol	MS,STD,LRI	18.17	1471	1468	-	40.09 ± 0.46 b	44.14 ± 0.90 b	139.11 ± 0.52 a	***
24	2-ethyl-1-hexanol	MS,STD,LRI	18.90	1504	1504	0.33 ± 0.13	-	0.32 ± 0.09	0.38 ± 0.24	ns
25	1-octanol	MS,STD,LRI	20.36	1573	1572	0.21 ± 0.09	0.37 ± 0.21	0.48 ± 0.35	0.32 ± 0.17	ns
26	(Z)-2-Octen-1-ol ^2^	MS,LRI	21.60	1635	1620	0.05 ± 0.02 b	0.16 ± 0.13 a	0.04 ± 0.02 b	0.02 ± 0.01 b	***
27	2,7-Octadien-1-ol	MS,STD,LRI	23.00	1705	1693	0.02 ± 0.01	0.02 ± 0.01	-	-	ns
28	Benzyl Alcohol ^2^	MS,LRI	26.89	1916	1902	-	1.32 ± 0.06 b	3.44 ± 0.84 a	1.24 ± 0.02 b	***
29	Phenyl-ethyl Alcohol ^2^	M,LRI	27.5	1951	1946	0.033 ± 0.002 c	0.033 ± 0.002 c	0.039 ± 0.01 b	0.045 ± 0.01 a	***
	Sum alcohols					11.08 ± 5.56 c	56.61 ± 20.91 b	59.85 ± 31.30 b	158.2 ± 26.73 a	***
	*Ketones*									
30	2,3-pentanedione	MS,STD,LRI	8.83	1080	1085	14.75 ± 4.02 a	11.65 ± 3.20 b	12.43 ± 2.52 ab	10.15 ± 2.50 b	**
31	2,3-octanedione ^3^	MS,LRI	15.31	1344	1328	115.76 ± 50.41 ab	147.81 ± 125.23 a	155.01 ± 143.27 a	49.57 ± 27.17 b	***
32	2-nonanone	MS,STD,LRI	16.81	1409	1405	2.32 ± 1.19 b	5.71 ± 4.35 a	5.35 ± 4.49 a	4.75 ± 4.08 b	*
33	(E,E)-3,5-octadien-2-one ^3^	MS,LRI	19.87	1550	1569	41.72 ± 31.55 b	68.61 ± 33.27 b	74.89 ± 37.29 ab	96.97 ± 70.64 a	*
34	2-undecanone	MS,STD,LRI	21.32	1621	1601	5.18 ± 1.99 b	12.83 ± 7.05 a	12.37 ± 11.34 a	8.43 ± 5.12 ab	*
	Sum of ketones					179.71 ± 37.83 b	246.60 ± 99.26 a	260.05 ± 104.61 a	179.87 ± 60.77 b	*
	*Furans*									
35	2-ethylfuran	MS,STD,LRI	8.13	955	949	-	20.5 ± 5.27 c	30.43 ± 5.29 b	50.65 ± 0.68 a	***
36	2-pentylfuran	MS,STD,LRI	12.95	1245	1249	-	7.47 ± 5.59 b	7.63 ± 5.01 b	10.01 ± 1.02 a	***
	Sum of furans					-	27.98 ± 7.09 c	38.16 ± 11.15 b	60.66 ± 7.98 a	***
	*Acids*									
37	Acetic acid	MS,STD,LRI	18.43	1482	1479	-	0.12 ± 0.06 b	0.11 ± 0.07 b	0.20 ± 0.13 a	*
38	2-methyl-propanoic acid ^4^	MS,LRI	20.79	1594	1561	0.12 ± 0.05 c	0.10 ± 0.03 c	0.36 ± 0.05 b	0.48 ± 0.11 a	**
39	Pentanoic acid	MS,STD,LRI	22.80	1695	1720	0.57 ± 0.10 c	1.09 ± 0.10 b	0.63 ± 0.12 c	1.31 ± 0.11 a	***
40	Hexanoic acid	MS,STD,LRI	26.10	1871	1849	0.06 ± 0.01	0.06 ± 0.01	0.06 ± 0.01	0.05 ± 0.02	ns
41	Heptanoic Acid	MS,STD,LRI	27.97	1978	1963	0.07 ± 0.003 b	0.19 ± 0.13 a	0.08 ± 0.008 b	0.22 ± 0.12 a	***
	Sum of acids					0.82 ± 0.14 c	1.56 ± 0.31 b	1.24 ± 0.21 b	2.26 ± 0.26 a	***
	*Aliphatic Hydrocarbons*									
42	2,6,7-trimethyldecane ^5^	MS,LRI	8.62	1071	1058	0.23 ± 0.19 a	0.08 ± 0.05 b	0.11 ± 0.07 ab	0.04 ± 0.03 b	**
43	dodecane	MS,STD,LRI	11.70	1194	1200	30.34 ± 6.00	51.15 ± 41.38	32.43 ± 10.63	39.44 ± 13.82	ns
44	D-Limonene	MS,STD,LRI	12.10	1211	1197	52.21 ± 29.13	52.97 ± 25.61	41.62 ± 18.70	35.66 ± 14.75	ns
45	tridecane	MS,STD,LRI	14.29	1300	1300	4.83 ± 2.81 b	14.27 ± 7.84 a	13.96 ± 11.78 a	7.75 ± 6.23 ab	**
46	tetradecane	MS,STD,LRI	16.48	1394	1400	0.13 ± 0.05 c	0.51 ± 0.38 a	0.32 ± 0.22 a	0.13 ± 0.04 c	***
47	pentadecane	MS,STD,LRI	18.80	1499	1500	10.25 ± 8.30 c	44.39 ± 35.13 a	33.57 ± 28.59 ab	17.96 ± 12.85 bc	**
48	2,6,10,14-tetramethyl-pentadecane ^5^	MS,LRI	22.15	1662	1655	0.22 ± 0.15 b	0.72 ± 0.45 a	0.62 ± 0.54 ab	0.57 ± 0.25 ab	*
49	Z,Z,Z-4,6,9-nonadecatriene ^5^	MS,LRI	26.69	1904	1934	0.38 ± 0.32 b	0.48 ± 0.20 b	0.51 ± 0.32 b	0.95 ± 0.36 a	***
	Sum of aliphatic hydrocarbons					98.59 ± 39.01 b	164.58 ± 98.06 a	123.14 ± 61.74 ab	102.51 ± 40.68 b	***
	*Aromatic hydrocarbons*									
50	Ethylbenzene	MS,STD,LRI	10.37	1142	1128	-	9.632 ± 0.95 a	9.62 ± 1.08 a	2.60 ± 0.06 b	***
51	1,3-dimethyl-Benzene	MS,STD,LRI	10.58	1150	1143	-	1.15 ± 0.91	1.09 ± 0.85	1.14 ± 0.87	ns
52	p-Xylene	MS,STD,LRI	10.82	1160	1125	-	0.75 ± 0.23	0.84 ± 0.28	0.65 ± 0.18	ns
53	Styrene	MS,STD,LRI	13.9	1256	1254	-	1.77 ± 0.18 b	2.94 ± 0.12 a	1.08 ± 0.34 b	***
54	Trimethylpyrazine	MS,STD,LRI	17.3	1431	1437	0.12 ± 0.03 b	0.12 ± 0.03 b	0.14 ± 0.05 b	0.25 ± 0.08 a	***
	Sum of aromatic hydrocarbons					0.12 ± 0.02 c	13.42 ± 0.95 a	14.64 ± 1.81 a	5.72 ± 0.67 b	***

^1^ Data are expressed as µg of hexanal equivalents/kg. ^2^ Data are expressed as µg of 2-octen-1-ol equivalents/kg. ^3^ Data are expressed as µg of 2,3-pentanedione equivalents/kg. ^4^ Data are expressed as µg of pentanoic acid equivalents/kg. ^5^ Data are expressed as µg of tetradecane equivalents/kg. Mean values in the same column with different letters are significantly different (ns = not significant, * = *p* < 0.05, ** = *p* < 0.01, *** = *p* < 0.001). ^a^ Comparison with MS spectra obtained by NIST 0.5 library (MS), comparison with retention time and spectra of commercial standard (STD), comparison with linear retention indices (LRI) for an HP-FFAP capillary column, calculated by an n-alkanes (C7–C40) standard. ^b^ Retention time expressed as minutes. ^c^ Data are expressed as mg/kg.

## Data Availability

All the data are contained within the article and Supplementary Materials.

## Supplementary Materials

The following supporting information can be downloaded at: https://www.mdpi.com/article/10.3390/foods14040592/s1, Figure S1: Illustration of the coefficient distribution under null hypothesis obtained using permutation process.
