# Supplementary material for: Effect of Industrial Processing on the Volatile Organic Compound Fingerprint of Dry-Cured Tuna
Source: Foods. 2025 Feb 11;14(4):592. doi: 10.3390/foods14040592 (PMC11853805; doi:10.3390/foods14040592)
Supplement: Supplementary file 1 [file foods-14-00592-s001.zip › foods-3468734-supplementary.pdf]

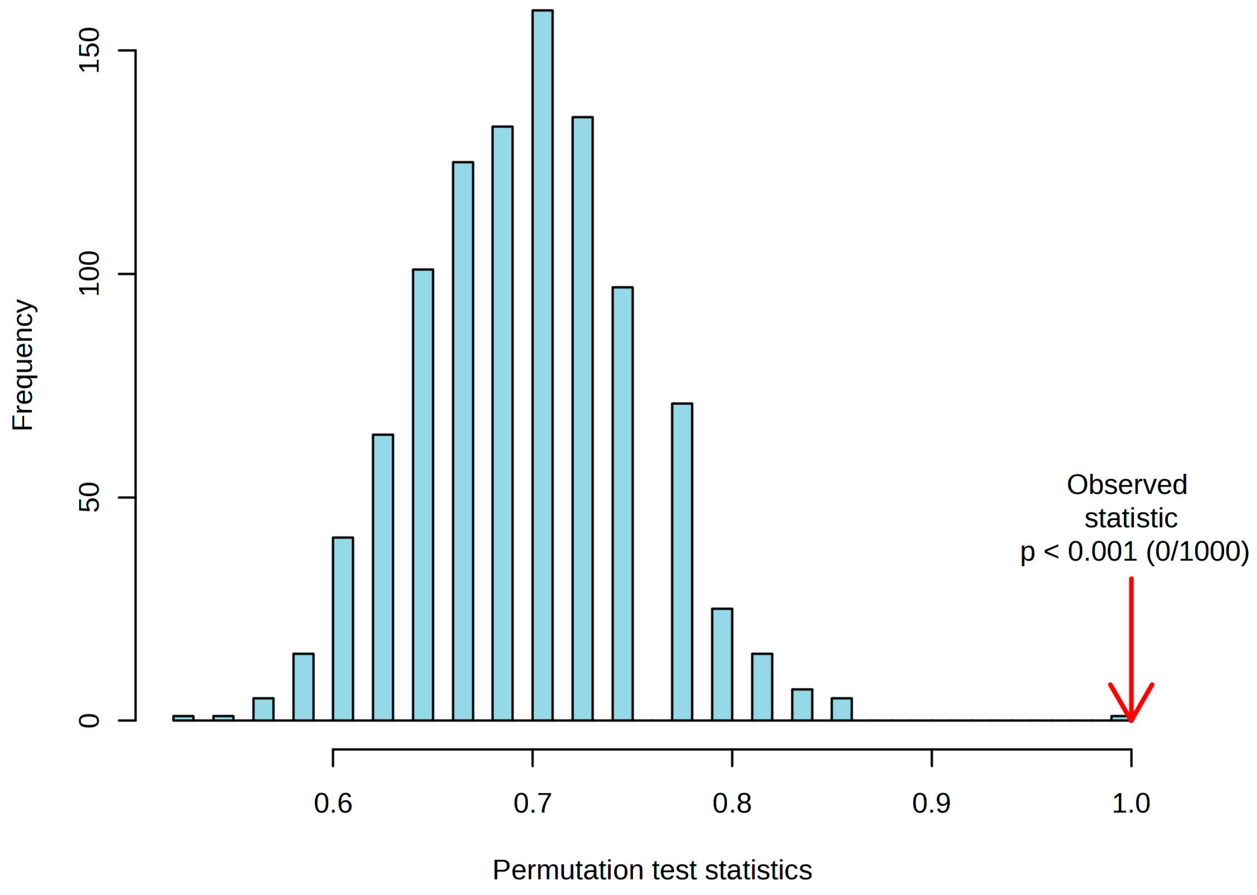

Figure S1. Illustration of the coefficient distribution under null hypothesis obtained using permutation process.
